# Agarose cell block technique as a complementary method in the diagnosis of fungal osteomyelitis in a dog

**Published:** 2012-04-24

**Authors:** D.S. Zanoni, F. Grandi, D.Q. Cagnini, S.M.G. Bosco, N.S. Rocha

**Affiliations:** 1 *Laboratory of Investigative and Comparative Pathology, School of Veterinary Medicine and Animal Science, Univ. Estadual Paulista – UNESP. Botucatu, Brazil*; 2 *Department of Pathology, Botucatu Medical School, Univ. Estadual Paulista – UNESP. Botucatu, Brazil*; 3 *Department of Microbiology and Immunology, Biosciences Institute, Univ. Estadual Paulista – UNEP, Botucatu, Brazil*

**Keywords:** Aspergillosis, Bone, Cell Block, Cytology, Dog

## Abstract

A 7-year-old Labrador Retriever female dog presenting left forelimb lameness for one day was admitted to the Veterinary Hospital (UNESP-Botucatu) for clinical evaluation. Several tests, including blood and image analysis, microbiological culture and cytology of lytic areas of affected bone were made in order to establish a diagnosis. Serum biochemical profile revealed increased levels of liver enzymes, plasma globulin, creatine kinase (CK) and calcium. Hemogram revealed anemia and leukocytosis; left humerus image analysis revealed an osteolytic lesion and cytology revealed a suppurative periostitis. Differential diagnosis was a nonspecific infectious inflammatory process or osteosarcoma. Since it was not possible to achieve a definitive diagnosis and there was a highly suspicious for an infectious agent, an agarose cell block of the bone marrow fine-needle aspiration was made. The cytological examination of cell block presented similar findings as described previously. However, additional stains including periodic acid-Schiff (PAS) were positive for fungal hyphae, which rendered a diagnosis of fungal osteomyelitis due to *Aspergillus* spp. This case report illustrates an uncommon cause of osteomyelitis for breed that was diagnosed by an underused method in veterinary medicine.

## Introduction

Fine needle aspirate cytology (FNAC) has been long used as a diagnostic tool (Powers, 1998; Magalhães *et al.*, 2001). Cytology offers a fast and cheap diagnostic method which is useful in cases of bone injuries. It has been used mainly for diagnosis of primary and secondary neoplastic bone lesions (Agarwal *et al.*, 1997; Jorda *et al.*, 2000).

FNAC can also help in the diagnosis of infectious bone diseases adding crucial information to the establishment of a timely diagnosis (Woods and Walker, 1996). The majority of opportunistic bone diseases in the dog are caused by fungi, mainly *Aspergillus* spp. (Butterworth *et al.*, 1995; Harkin, 2003).

Sometimes cytology does not provide sufficient information and the risk of false negative or undetermined diagnoses always exist (Kulkarni *et al.*, 2000; Handa *et al.*, 2005). For overcoming these problems and increase the yield of cytology samples, the technique of cell block was originally proposed for processing samples for electron microscopy (Yuan and Gulyas, 1981; Kulkarni *et al.*, 2000). The cell block technique increased the chance to achieve a positive result and also helped to demonstrate better architectural patterns which could be of great help in the routine for an approaching correct diagnosis (Mansy *et al.*, 2006; Thapar *et al.*, 2009).

The aim of this case report is to describe the use of cell block technique in the diagnosis of fungal osteomyelitis in a dog.

## Case Details

A 7-year-old spayed female Labrador Retriever presenting left forelimb lameness for one day was admitted to the Veterinary Hospital (UNESP-Botucatu) for clinical evaluation. The animal had a clinical history of recurrent lameness and intermittent reluctance to move over a period of one year.

The initial treatment included tramadol (4 mg/Kg TID), firocoxib (5 mg/Kg SID) and clavulanic acid plus amoxicilin (25 mg/Kg BID).

Blood analysis revealed an increase in lactate dehydrogenase (381.49 IU/L; reference range [RR] = 45-233 IU/L), alkaline phosphatase (263.6 UI/L; [RR] = 20.00-156.00 UI/L), total serum protein (10.8 g/dL; [RR] = 5.40-7.10 g/dL), total plasmatic protein (8.2 g/dL; [RR] = 6-8 g/dL), globulin (7.50 g/dL; [RR] = 2.70-4.40 g/dL), CK (63.4 UI/L; [RR] = 1.15-28.40 UI/L) and calcium (16.0 mg/dL; [RR] = 9.00-11.30 mg/dL).

Hemogram revealed mild anemia (5.0 x 10^6^/µL; [RR] = 5.50-8.50 x 10^6^/µL), PCV (33%; [RR] = 37-55%), increased MHC (40%; [RR] = 32-36%), leukocytosis (18.1/µL; [RR] = 6.00-17.00/µL) with neutrophilia (15390/µL; [RR] = 3000-11500/µL), mild lymphopenia (900/µL; [RR] = 1000-4800/µL) and monocytosis (1800/µL; [RR] = 150-1350/µL).

**Fig. 1:**
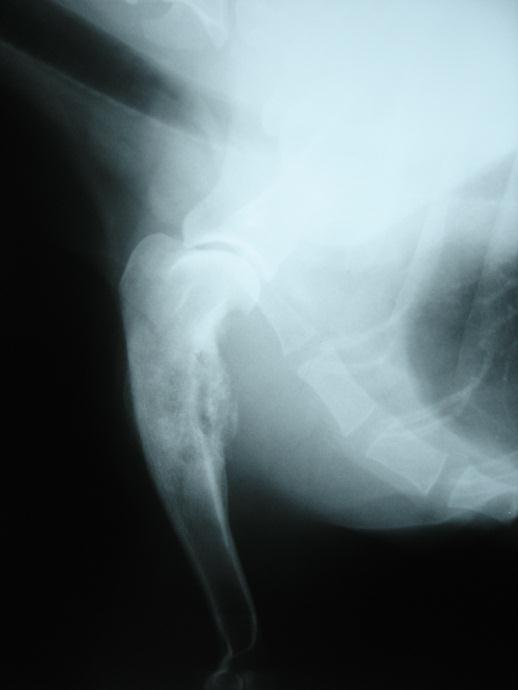
Radiography reveals an osteoproliferative and osteolytic lesion in the metaphysis of the left humerus. Mediolateral view.

Radiography revealed an osteoproliferative and osteolytic lesion in the metaphysis of the left humerus (Figure 1).

Periosteal samples of the affected limb were obtained by ultrasound guided fine-needle aspiration for cytopathologic evaluation. The collected material was spread on histological slides, air-dried, methanol fixed and Giemsa stained.

Smears showed high cellularity with degenerate and non-degenerate neutrophils in a 3:1 ratio, fewer histiocytes and abundant cellular debris on an acidophilic background.

Rare osteoblasts, sometimes associated with a slight pink and amorphous material (bone matrix) were observed as well. No infectious agents were seen. Initial diagnosis was suppurative periostitis.

Three days after, the animal was submitted to bone biopsy and bone marrow aspiration in order to acquire a more representative sample. The bone marrow aspiration was submitted to the agarose cell block technique, bacteriological and fungal culture.

**Fig. 2:**
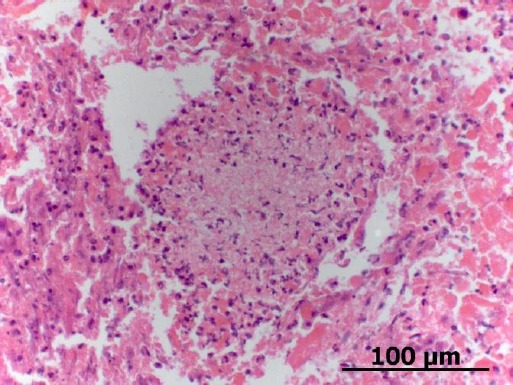
Agarose cell block section of a bone marrow cytology showing a necrotic center composed by cellular and nuclear debris. H&E stain, 400x.

**Fig. 3:**
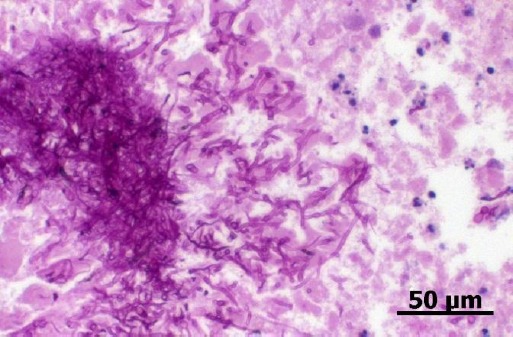
Agarose cell block section of a bone marrow cytology sample showing PAS positive septate hyphae. PAS stain, 400x.

The bone biopsy was fixated in 10% formalin and routinely processed. The samples for cell block of agarose were packed in an Eppendorf tubes, 70% ethanol fixed, and centrifuged at 3000 rpm for 10 minutes. The supernatant was removed and 2% liquid agarose was added. Then, the samples were re-centrifuged at 3000 rpm for 10 minutes to obtain a solid pellet. Finally, the pellet was paraffin embedded, processed for histopathological evaluation and stained with hematoxilin and eosin (H&E) (Figure 2) and periodic acid-Schiff (PAS) stain (Figure 3).

Bone biopsy presented moderate thinning of the bone matrix with diffuse distribution associated with the moderate amount of osteocytes, discrete presence of osteoblasts and rare osteoclasts, which established a final diagnosis of osteoporosis.

Cell block samples presented similar cytopathological previously described findings in addition to several PAS positive, hyaline, septate and regular hyphae with parallel walls and 45º branching angle, compatible with *Aspergillus* spp.

Microbiological culture yielded negative results. The final diagnosis was suppurative and mycotic osteomyelitis due to *Aspergillus* spp.

It was prescribed itraconazole (300 mg/Kg SID) for three months. The animal showed continuing improvement until the present time.

## Discussion

*Aspergillus* spp. is an opportunistic hyphomycete with a worldwide distribution that can cause localized and systemic infections in immunocompromised humans and dogs. It is commonly found in soil, water and organic matter (Harkin, 2003).

The diagnosis of aspergillosis can be challenging in many cases, because fungal structures are not always present in superficial cytological samples as seen in this case; thus several techniques should be applied to detected such agents (Harkin, 2003).

The main differential diagnosis in a middle-age dog of large breeds with lameness and forelimbs enlargement is skeletal osteosarcoma. The location and radiographic features described here were highly suspicious for a skeletal osteosarcoma (Burk and Ackerman, 1996).

However, due to the suppurative nature of cytological samples, leukocytosis with neutrophilia and increased levels of total protein and alkaline phosphatase we were unable to discard a possible infectious bone disease (Guerin *et al.*, 1993; Butterworth *et al.*, 1995; Harkin, 2003).

Bone marrow cell block analysis allowed us to confirm the fungal nature of the bone lesion. Since we could not detect any infectious agents using FNAC of periosteal surface and lytic areas, we opted to collect a deeper cytological sample (i.e. bone marrow) in addition to superficial bone cytology. Although microbiological culture yielded negative results we suspected that the etiological agent was *Aspergillus* spp. due to the presence of branching pattern (Koneman *et al.*, 2001).

Cell block technique is used mainly for cytological specimens from pleural, peritoneal effusions and bronchial washes (Nigro *et al.*, 2007), and was important in this case because it maintained the original fungal architecture as well as avoided more invasive techniques such as bone marrow biopsy.

Bone marrow biopsy probably also could achieve the same diagnosis obtained with cell block technique. However, the last constitutes in more invasive and laborious technique.

In conclusion, although rarely used in veterinary medicine, the cell block technique poses some advantages to the classical bone biopsy with less tissue invasion and offering a quick diagnosis; both are highly desired in this case. Furthermore, additional techniques such as PAS stain from cell block samples could be of great value in etiological diagnosis.
